# Appropriateness of Proton Pump Inhibitor Prescription Evaluated by Using Serological Markers

**DOI:** 10.3390/ijms24032378

**Published:** 2023-01-25

**Authors:** Michele Russo, Kryssia Isabel Rodriguez-Castro, Marilisa Franceschi, Antonio Ferronato, Maria Piera Panozzo, Lorenzo Brozzi, Francesco Di Mario, Pellegrino Crafa, Giovanni Brandimarte, Antonio Tursi

**Affiliations:** 1 Anesthesiology, Critical Care and Pain Medicine Division, Department of Medicine and Surgery, University of Parma, 43121 Parma, Italy; 2 Endoscopy Unit, Department of Medicine, ULSS7 “Pedemontana”, “Alto Vicentino” Hospital, 36014 Santorso, Italy; 3 Laboratory of Clinical Pathology, ULSS7 “Pedemontana”, “Alto Vicentino” Hospital, 36014 Santorso, Italy; 4 Department of Medicine and Surgery, University of Parma, 43121 Parma, Italy; 5 Division of Internal Medicine and Gastroenterology, “Cristo Re” Hospital, 00167 Rome, Italy; 6 Territorial Gastroenterology Service, ASL BAT, 76123 Andria, Italy

**Keywords:** proton pump inhibitors, gastric function, *Helicobacter pylori*, acid output, gastrin, pepsinogen, reflux disease

## Abstract

Inappropriate prescription of proton pump inhibitors (PPI) has been widely reported, often lacking initial exclusion of *Helicobacter pylori* (HP) infection and evaluation of gastric functional status. The aim of this study was to evaluate the utility of gastric functional tests to define the acid output, as well as HP status, in order to better direct PPI therapy prescription. Dyspeptic patients without alarm symptoms from a primary care population were evaluated. For each patient, serum Pepsinogen I (PGI) and II (PGII), gastrin 17 (G17) and anti-HP IgG antibodies (Biohit, Oyj, Finland) were determined. For each subject, data were collected regarding symptoms, past medical history of HP infection, and PPI use. Therapeutic response to PPIs was determined according to PGI and G17 values, where G17 > 7 in the presence of elevated PGI and absence of chronic atrophic gastritis (CAG) was considered an adequate response. Among 2583 dyspeptic patients, 1015/2583 (39.3%) were on PPI therapy for at least 3 months before serum sampling, and were therefore included in the study. Active HP infection and CAG were diagnosed in 206 (20.2%) and 37 (3.6%) patients, respectively. Overall, an adequate therapeutic response to PPIs was observed in 34.9%, reaching 66.7% at the highest dose. However, 41.1% and 20.4% of patients showed low (G17 1-7) or absent (G17 < 1) response to PPI, regardless of the dosage used. According to gastric functional response, most patients currently on PPI maintenance therapy lack a proper indication for continuing this medication, either because acid output is absent (as in CAG) or because gastrin levels fail to rise, indicating absence of gastric acid negative feedback. Lastly, HP eradication is warranted in all patients, and gastric function testing ensures this pathogen is sought for and adequately treated prior to initiating long-term PPI therapy.

## 1. Introduction

Proton pump inhibitors (PPIs) are first-choice drugs for the treatment of acid-related disorders, such as gastroesophageal reflux disease and peptic ulcer disease, due to their ability to effectively inhibit gastric acid secretion. PPI use and ensuing hypochlorhydria are associated with compensatory hypergastrinemia, which, in fact, was one of the initial concerns of long-term therapy [1].

PPIs are among the most widely prescribed drugs in the world, and their use is continuously increasing, especially for long-term treatment, mainly due to their effectiveness combined with a good safety profile. However, PPIs are being increasingly used in inappropriate scenarios, such as for unregistered indications and cases not in line with the NHS prescription criteria [2,3]. Inadomi et al. demonstrated that PPI use is largely inappropriate and costly, rendering strategies that allow for adequate selection of patients who actually benefit from therapy with these drugs a priority of health systems [4,5]. Multiple guidelines and campaigns promote discontinuation of PPIs when possible, usage of the minimum effective dose, and pursuing alternative diagnoses or treatments in patients whose symptoms do not respond to PPI therapy [6,7,8]. Although infrequent, affecting 1–3% of users, adverse events may range from headache, nausea, abdominal pain, constipation, flatulence, diarrhea, rash, and dizziness, while modification of gastric pH and its action on CYP P450 system may significantly alter drug absorption and metabolism [9,10]. Moreover, there is growing evidence that acid suppression increases the risk of enteric infections by *Clostridium difficile*, amongst other pathogens, and may contribute to bacterial overgrowth [11,12,13]. 

Determination of gastric function, including acid output and infection with HP, are useful tools to guide the clinician in evaluating gastric morphology and function, and represent an invaluable tool to guide prescription and/or suspension of PPIs, when appropriate [14,15,16]. The gastric function test constitutes a non-invasive determination of four serum parameters, interpretation of which is based on the combination of values included in the panel. These four biomarkers include pepsinogen I (PGI), pepsinogen II (PGII), amidated gastrin-17 (G17), and HP antibodies. The determination of different profiles provides accurate estimates of the capacity of the corpus and antrum mucosa to secrete gastric acid and G17, respectively, and identifies important gastric conditions, such as HP infection. As PGI and PGII increase with inflammation, and gastrin levels reflect integrity of antral mucosa and an intact gastric acid negative feedback mechanism, gastric function testing provides information on the grade and topography of atrophic gastritis, which in turn represent an increased risk of gastric cancer [17,18,19,20,21,22]. 

The aim of this study was to evaluate the usefulness and efficacy of PPI therapy using gastric function testing, identifying patients in whom a different therapeutic strategy is warranted, including patients with active HP infection, patients with chronic atrophic gastritis, or patients in whom response to therapy is very low or absent, in terms of G17 elevation. 

## 2. Results

A total number of 2583 of patients undergoing gastric function test were evaluated, 1015 of whom had been on PPI therapy for at least 3 months prior to examination, and thus constituted the study population. Table 1 summarizes the demographic characteristics, risk factors related to lifestyle, and possible hereditary conditions relevant to gastrointestinal pathology in the study population. 

**Figure 1:**
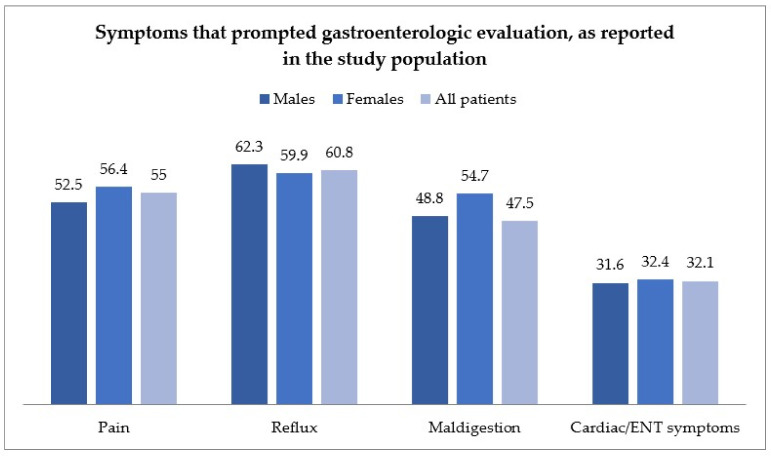
Symptom distribution in the study population, according to gender, and grouped by the following categories: pain-related, reflux-related, maldigestion-related, and cardiac or ear-nose and throat (ENT)-related. ENT: Ear, nose and throat.

Figure 1 reports the symptoms of evaluated patients upon inclusion in the study, grouping patient complaints in general categories, as detailed above. 

Most patients complained of more than one symptom, and symptoms were distributed as follows: 55% of patients complained of pain (abdominal or chest pain); 60.8% of patients complained of reflux; 47.5% of symptoms of maldigestion; and 38.6% of cardiac or ENT (Ear, nose and throat) symptoms. The main endoscopic and histological findings are reported in Supplementary Table S1. 

Overall, mean values of PGI, PGII, PGI/PGII, G17 and anti-HP IgG were 139.1 μg/L, 12.8 μg/L, 12.4, 11.7 pmol/L and 25.3 EIU, respectively. No statistical differences were observed between genders, with the exception of G17, which was significantly higher in females with respect to males (13.6 ± 23.9 vs. 8.4 ± 14.6, respectively; *p* = 0.0001). 

**Figure 2:**
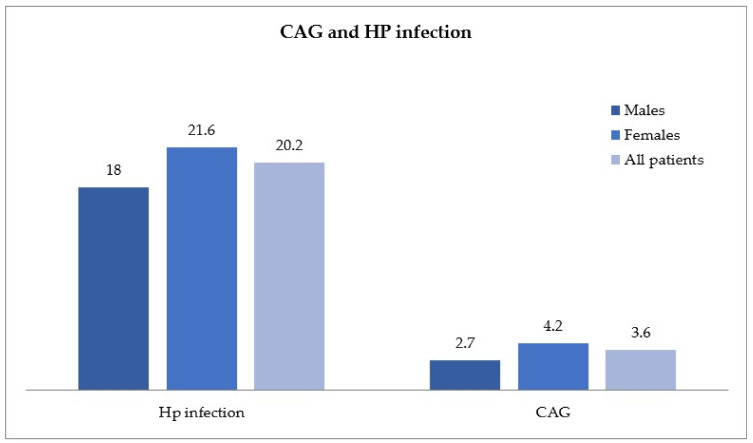
*Helicobacter pylori* (HP) infection and chronic atrophic gastritis (CAG) in the study population, distributed by gender.

A total of 206 patients (males n = 68, females n = 138), were diagnosed with active HP infection and were significantly younger (mean age 49.2 ± 14.7 years) than the 37 patients identified as CAG (males n = 10, females n = 27), with a mean age of 62.9 ± 17.3 (*p* < 0.05), as shown in Figure 2. A high concordance between elevated levels of G17, low levels of PGI (NPV, Negative Predictive Value, ~98%) and the histological report of CAG was found. 

Table 2 shows the response rate to PPI therapy assessed using G17, according to PPI therapy dosing, both in CAG and non-CAG patients, while Table 3 shows response rates to PPI, as assessed by G17 levels, correlated to overall gastric function parameters. 

PGI levels correlated with PPI efficacy, increasing parallel to G17 elevation (194 ug/L, 127 ug/L and 91.2 ug/L in adequate-, low-, and non-responders, respectively (*p* < 0.001).

**Figure 3:**
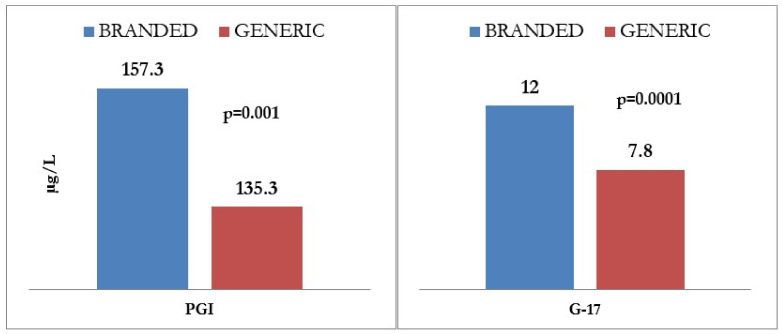
Correlation between efficacy of generic vs. branded proton pump inhibitor (PPI) therapy on gastric function, as determined by gastrin 17 (G17) and prostaglandin I (PGI) levels. G17: gastrin 17; PGI: prostaglandin I.

A significant difference regarding response rates was observed between generic and brand drugs (*p* = 0.021), as shown in Figure 3, the latter being associated with a higher proportion of adequate responders and a lower proportion of non-responders. The proportion of low-responders, however, was similar for both branded and generic drugs. 

Finally, Table 4 shows the logistic regression analysis for the individual factors that may influence the therapeutic response to PPIs, dichotomized as no-response (G17 ≤ 1 vs. response G17 > 1, which correlates with older age, female sex, the presence of active HP infection, and use of full dose and branded PPIs.

## 3. Discussion

PPIs are highly effective for a wide range of acid-peptic conditions, but the evidence suggests they are being excessively prescribed. Inappropriate scenarios include drug prescription for longer duration and at higher doses than advised, according to current guidelines. At a time of growing concern over rising drug costs and limited healthcare resources, potentially inappropriate or unnecessary use of expensive drugs like PPIs should be limited where possible. In contrast to conditions such as hypertension, anticoagulation, or diabetes, in which therapeutic strategies are adopted according to specific laboratory or clinical targets, PPI use has been exclusively symptom-driven and their efficacy has not been evaluated with objective parameters. On the contrary, evaluation of gastric function best serves both patients and future prescription of adequate therapy, which will then be tailored according to an initial functional evaluation. The relative safety, widespread availability, and general unawareness of the possibility of evaluating gastric function using non-invasive tests is probably the basis for excessive PPI use. Most of the scientific literature on serological gastric markers has focused on their use in the diagnosis of gastroesophageal reflux disease and CAG. Although some studies have evaluated the influence of PPIs on PGI and G17 [23,24], their use as a first-level test in evaluating the prescriptive appropriateness of PPIs remains a largely unexplored topic.

The main purpose of this study was to evaluate whether the use of a serological, non-invasive test may be of value before initiating PPI therapy in the daily clinical practice of an outpatient setting. In agreement with scientific literature, we found a high concordance between elevated levels of G-17, low levels of PGI, and the histological report of CAG; moreover, it is well known that the use of anti-HP IgG in combination with high levels of PGII allows to discriminate current infections from previous infections with high sensitivity (95%) and good specificity (83%) [14,18,19,25,26,27].

As shown above, 18.2% of the patients enrolled in the study were smokers, without a significant difference between males and females; alcohol consumption (at least 1–2 units/day) was reported in 47.5%, and was notably higher in men than women (*p* = 0.0001). Autoimmune thyroiditis was present in 13.4%, with higher prevalence in women than in men (*p* = 0.0001); patients who had a previous HP eradication represent 20.4% of the total, with a significant difference between men and women (*p* = 0.002).

Patients with familiarity for gastric cancer were 10.5% and, as expected, there was no difference between sexes. Aspirin therapy was reported in 7.4% of patients, whereas 19.4% of patients reported being on NSAIDs therapy during the 3 months prior to study enrollment (higher consumption in women than men; *p* = 0.007).

Of note, female gender is significantly overrepresented in the study (62.9%), with a mean age which is significantly higher (47.9 ± 15.7 vs. 45.0 ± 14.8, *p* < 0.05) with respect to males on PPI therapy. This observation could respond to a more elevated frequency of PPI prescription in females in general, which has been reported by other researchers. Tosetti et al. found that, in the general practice in a population of Europeans, patients who are more frequently prescribed PPIs are generally older, female, and more frequently diagnosed with gastroesophageal reflux disease, gastric or duodenal ulcers, arthropathy, heart disease, and cancer than the rest of the population [28]. Moreover, PPI users more frequently receive prescriptions for non-steroidal anti-inflammatory drugs, acetylsalicylic acid, oral anticoagulant therapy, and systemic steroids [29,30]. In addition, another possible explanation for female overrepresentation in the study group is the fact that not all patients who are on chronic PPI therapy are evaluated by gastroenterologists, and the evaluation by the latter could be more often requested by family physicians or other specialists driven by poor symptom control on PPIs. In this scenario, it is possible that more female patients are referred for evaluation with gastroenterologists because of non-responsiveness to PPI therapy due to the fact that many of such patients, especially females, could be affected by CAG. 

The elevation of G17 translates a reduction in acid output, which stimulates negative feedback-regulated gastrin production in the antrum, but also in the pancreas and duodenum. Variations in G17 levels were therefore used as an indicator of therapeutic response to PPIs, which, depending on their efficacy, exert an impact on acid output reduction, which is then reflected on the elevation of gastrin levels. In this study, PGI reveals itself as another useful parameter to evaluate PPI efficacy. In fact, PGI levels parallel the increase in G17, decreasing from mean values of 194 ug/L in adequate responders to 91.2 ug/L in non-responders. Possibly, PGI elevation reflects the PPI-induced, gastrin-mediated, trophic effect on gastric mucosa that ensues after acid suppression. Moreover, it is possible that elevated levels of PGI single out a group of high acid output patients, refluxers, who benefit from PPI therapy. 

In our study, all infections found with serology were then confirmed by biopsies, and 20.3% of patients had active HP infection. Considered a class I carcinogen by the IARC [31], HP infection warrants eradication, which underlines the importance of testing for this pathogen before starting PPI therapy, not only to halt carcinogenesis, but also because PPI therapy could yield falsely negative results in some HP testing techniques (i.e., urea breath-test) [32,33].

A diagnosis of CAG was established in 3.6% of patients on PPI therapy, which clearly represents an inappropriate indication for PPI, since, by definition, in the presence of CAG the stomach does not produce acid, making the use of PPIs useless and potentially harmful, exposing patients to the side effects of drugs for no reason. The female population is overrepresented in the CAG subgroup, although this difference did not reach statistical significance. Additionally, autoimmune thyroiditis, which is correlated to other autoimmune diseases, especially autoimmune gastritis, was more frequent in women, suggesting that the higher frequency of CAG in women might be explained at least in part by the presence of autoimmune disease. Similarly, levels of G17 were significantly higher in females with respect to males. Although CAG was more frequent in females, this does not account for the observed difference in G17 between genders. A possible explanation is that G17 acts as an early marker of autoimmunity, identifying a subgroup of patients that, in time, will go on to develop a more florid clinical picture of autoimmune gastritis.

Regarding therapeutic efficacy of PPIs, an adequate therapeutic response was observed in 34.9%, reaching 66.7% at the highest dose. However, 41.1% and 20.4% of patients showed a low (G17 1-7) or absent (G < 1) response to PPI, regardless of the dosage used. A global response rate of 34.9% can arguably be considered disappointingly low, considering the fact that PPIs are often regarded as efficacious drugs. Notably, in spite of showing lack of response to PPI, 20.4% receive this drug daily, and the proportion of patients with inappropriate PPI prescription rises even more when CAG patients are considered as well. The proportion of patients on half, full, and high dosing is not distributed differently amongst non-responders, intermediate-responders, and responders, suggesting that the response to PPI may be dose-independent. Cytochrome P450 isoenzyme 2C19 genotype differences may account, at least in part, for such variability in response rates that are unrelated to dosing, although this determination may be unfeasible in clinical practice [34]. Brand vs. generic drug variants might also account for some of the observed differences in response rates, as high-, full- and half-dose groups were each composed of patients on brand or generic drugs, and the group size did not allow for statistically significant differences to emerge between them [35]. Other causes of different response rates may include lack of compliance to therapy, incorrect drug intake, and different bioavailability between generic and branded PPIs. In the present study, the therapeutic response of PPIs correlated with several factors, including older age, female sex, the presence of HP infection, and use of full dose and branded PPI at multivariate analysis. Interestingly, having eradicated HP infection does not correlate with better response rates. 

In this study, we have found that patients on branded drugs therapy showed a better therapeutic response than patients on generic drugs therapy, which Otten et al. suggest can be due to: (1) biphasic metabolism, where the raised pH in the stomach may prematurely inactivate the PPI, with an unpredictable effect; (2) differences in acid-resistant coating of the generic products; and (3) the influence of multiple dosing of PPIs after several days’ use [36]. The authors conclude that all three factors may contribute to the difference in absorption and therefore clinical effectiveness, but, in our opinion, further studies are needed, such as possibly double-blind controlled clinical trials comparing branded and generic PPIs to shed light on the issue.

Regardless of the underlying reason for poor response to PPI, gastric function testing allows its rapid and quantifiable determination, identifying patients who will not benefit from continuation of therapy with the same drug. Furthermore, a possibility that can be explored readily is the switching of drugs, be it from generic to brand, or between generics, or to a different molecule, whenever the first showed no or poor response [37]. Gastric function testing would be of value in this scenario to guide therapeutic decisions, not only to suspend ineffective therapy (i.e., presence of CAG), but to eventually tailor PPI therapy to the single patient. More importantly, GastroPanel is of utmost value prior to starting PPI therapy, in order to rule out mainly two scenarios where PPI therapy (as monotherapy) is useless: the presence of CAG (where acid output is already abated) and HP infection (where specific antibiotic therapy is warranted). 

The presence of HP infection could theoretically hamper the Gastropanel’s capacity of quantifying the effectiveness of PPI therapy, by causing an elevation of G17 and PGII, and altering the levels of PGI. However, when the subpopulation of patients with active HP infection were removed from the analysis, results did not vary, and all variables retained their statistical significance. 

There are some limitations of this retrospective, observational study. The data collection sheet does not allow for specification of whether several types of drugs (either different molecules or different brand/generic drugs) have been used in the same patient. This could partly explain the high percentage of non-responders to therapy; on the other hand, this data probably better expresses what happens in real life, and the use of this serological test has proved to be a very reliable tool in identifying these patients, whether they are non-compliant with therapy, have not received adequate instructions on the correct use of these drugs, or are fast metabolizers. Another limitation lies in the small size of the subgroups of different response categories, which renders the further division into branded vs. generic drugs void of statistical significance. A larger study would be necessary to more accurately establish differences in measured PPI response according to brand vs. generic drugs at the different dosing schemes. Moreover, one of the limits of the present study is the lack of statistically determined cut-off values for G17 determination as a discriminant between no-, low- or adequate-responders, as values were arbitrarily selected. Finally, another limitation of the study is the fact that basal levels of studied parameters (G17, PGI, PGII), while off PPI therapy, were not obtained. A study including basal levels and comparing them to post-PPI therapy levels would allow for a finer tuning in identifying acid refluxers and establishing possible causes of non-response or low response.

## 4. Materials and Methods

This retrospective study considered consecutive patients evaluated in a tertiary hospital in Northeastern Italy from 2015 to 2018 for dyspepsia without alarm symptoms. Records of patients evaluated with gastric function testing were retrieved and matched to histological reports of biopsy samples taken during upper gastrointestinal endoscopy (data available as Supplementary Materials). At our center, evaluation of gastric function requires informed compilation of a form, which includes consensus for using data anonymously. The following variables were thus collected and subsequently analyzed: age; gender; body mass index; past medical history of cigarette smoking and alcohol intake; clinical indications for evaluation; presence/absence of gastrointestinal and nonspecific symptoms, including chest pain, persistent cough, and persistent need for throat clearing; past/present medical history of PPI use; past/present medical history of therapy with NSAIDs and/or aspirin; family history of gastric ulcer or neoplasm; past medical history of autoimmune diseases, including CAG; and previous diagnosis of HP infection.

### 4.1. Inclusion Criteria 

The inclusion criteria were as follows:

- Age > 18 years.
- Determination of gastric function using serological testing.
- PPI use for at least 3 months prior to evaluation. Therapy with PPI included the following generic and brand drugs: Omeprazole (Antra^®^, Omeprazen^®^, Mepral^®^, and Losec^®^), Esomeprazole (Nexium^®^, Lucen^®^, Axagon^®^, and Esopral^®^, Pantoprazole (Pantorc^®^, Pantopan^®^, Pantecta^®^, Peptazol^®^), Rabeprazole (Pariet^®^), and Lansoprazole (Zoton^®^, Limpidex^®^, and Lansox^®^).
- The presence of symptoms attributed to gastroesophageal acid-related disease, for which patients were on PPI therapy; symptoms were assigned to the following categories: pain symptoms (including epigastric and abdominal pain); reflux symptoms (including heartburn, acid regurgitation, persistent hiccup, belching, sialorrhea, and globus pharynges); maldigestion symptoms (including nausea, bloating, flatulence, rumbling abdomen, bitter taste in the mouth, and aerophagia); otorhinolaryngological symptoms (including hoarseness, chronic laryngitis, coughing, glossitis, and pharyngitis); and chest/cardiac symptoms (including thoracic pain, tachycardia, extrasystoles, and dyspnea).

### 4.2. Exclusion Criteria

Subjects with the following conditions, which may alter gastric function and warrant a different diagnostic approach, were excluded:

- Alarm symptoms or signs as an indication for upper endoscopic and/or gastric functional evaluation, such as weight loss, anemia, presence of bright red blood per rectum, coffee-ground stools, ematochezia, dysphagia, persistent vomiting or vomiting of coffee-ground material or hematemesis, and persistent diarrhea.
- History of Zollinger-Ellison syndrome.
- Pyloric stenosis.
- Previous surgery of the esophagus and/or gastrointestinal tract (except for appendectomy and cholecystectomy).
- Malignant gastrointestinal tumors.
- Therapy with H2-blockers.

### 4.3. Serological Evaluation of Gastric Function 

A 5 mL of blood sample was drawn from each patient after 12 h of fasting. The sample was centrifuged for 10 min at a 3000-rpm speed. The obtained serum was used for the determination of PGI, PGII, G17 and IgG anti-HP. A specific Biohit (GastroPanel^®^, Biohit Oyj, Helsinki, Finland) kit was used based on an enzyme immunoassays quantitative investigation: Enzyme Linked Immunosorbent Assay (ELISA). Detailed information on sample processing is available as Supplementary Materials. Reference values, as validated in multiple studies are as follows: 30–100 µg/L for PGI, 2–15 µg/L for PGII, 1–10 pmol/L for G17, and <30 U/L for IgG anti-HP. Serological criteria for CAG diagnosis included PGI <30 µg/L, G17 >14 pmol/L, and a low PGI:PGII ratio. Diagnosis of active HP infection was based on high levels of PGII (>10 µg/L), a marker of gastric inflammation, and on positivity for IgG antibodies anti HP (>30 U/L). The combination of the aforementioned parameters allowed the definition of three distinct serologic profiles: (a) subjects with active HP infection; (b) subjects with CAG; and (c) subjects with neither HP infection nor CAG. The third group of patients represent subjects that presumably have high acid output, and in whom PPI therapy could target symptom alleviation. 

### 4.4. Endoscopic and Histological Evaluation

In brief, patients with a positive finding of anti HP antibodies or with PGI/PGII ratio <3 underwent upper gastrointestinal endoscopy with routine biopsies of the gastric mucosa, with subsequent histological evaluation to confirm the diagnosis of HP infection or CAG, as detailed in the Supplementary Materials section. 

### 4.5. PPI Therapy Therapeutic Response

All formulations of PPIs currently used by the study population (omeprazole, lansoprazole, pantoprazole, rabeprazole, esomeprazole), both branded and generic, were considered. Patients were divided into groups according to dosing: half dose, full dose or high dose, as shown in Table 5. The therapeutic response of PPIs was evaluated using G17 levels as the index parameter; values >7 pmol/L, 1–7 pmol/L, and <1 pmol/L were considered as adequate, low, or no response, respectively. Cut-off values were established based on previously published data. Employing the logistic regression analysis, PPI response was evaluated dicotomically (Responder/Non responder) using a cut-off value of G17 > 7.

### 4.6. Statistical Analysis

Statistical analysis was performed with the SPSS statistical software program for Windows (version 20.1). The analysis of the differences between groups in the event of normal data distribution was performed by means of one-way analysis of variance (ANOVA). In the event that the verification of normal data distribution was negative, the Kruskall–Wallis test was used. The study of differences between dichotomous qualitative groups and variables was carried out using Pearson’s chi-square test. Data were expressed as mean ± standard deviation (DS) for qualitative variables and as a percentage of the total for quantitative ones. All *p* values were two-tailed with statistical significance indicated by a value of *p* < 0.05. The Spearman correlation test was used to analyze the association between PGI levels and response to PPI in terms of G17 elevation. All variables with a *p* > 0.10 were considered for inclusion through a manual forward stepwise variable selection process in a multivariable logistic regression analysis. 

### 4.7. Ethics

This study was conducted in accordance with the Declaration of Helsinki, and approved by the local Ethics committee of the ULSS7 “Pedemontana”, with the protocol number 92687. According to the study protocol, patients who did not give their consent at the time of the gastric function testing were excluded. 

## 5. Conclusions

The low overall PPI response rate suggests that clinicians must come to terms with the fact that a large number of patients with symptoms of presumably upper gastrointestinal nature have actually no connection with excessive acid production, and therefore a need for reassessment of PPI prescription is warranted. This study revealed that the use of a panel composed of four serological markers (PGI, PGII, G17, anti-HP IgG) is a valid tool to be used as a first-level test to be carried out before undertaking a therapy with PPIs, to evaluate the state of the mucosa, HP infection and, more generally, the prescribing appropriateness of these drugs. Such a strategy may be adopted within the diagnostic algorithms and in prescribing guidelines, avoiding unnecessary and harmful side effects related to PPI therapy or to invasive diagnostic procedures. Nonetheless, the use of pre-emptive gastric function testing may lead to important burden reduction in the healthcare system.

## Figures and Tables

**Table 1:** Demographic and clinical characteristics of patients on proton pump inhibitor (PPI) therapy, according to gender.

	Males	Females	*p*	Total
**Patients (n, %)**	377 (37.1)	638 (62.9)		1015 (100)
**Mean age ±SD (years)**	45.0 ± 14.8	47.9 ± 15.7	0.003	46.8 ± 15.4
**Range age (years)**	18–84	18–95		18–95
**Body mass index (kg/m^2^)**	24.9 ± 3.6	23.9 ± 4.7	0.004	24.3 ± 4.4
**Cigarette smoking (%)**	18.6	17.9	0.061	18.2
**Daily alcohol intake (%)**	64.3	37.7	0.0001	47.5
**Autoimmune thyroiditis (%)**	3.4	19.3	0.0001	13.4
**HP eradication (%)**	15.6	23.2	0.002	20.4
**Gastric cancer familiarity (%)**	10.1	10.8	0.454	10.5
**NSAIDs use (%)**	15.4	21.8	0.007	19.4
**Aspirin use (%)**	8.8	6.6	0.125	7.4

NSAIDs: Non-steroidal antinflammatory drugs.

**Table 2:** Response rate to proton pump inhibitor (PPI) therapy assessed using gastrin 17 (G17), according to PPI dosing, in both patients with and without CAG.

		PPI Therapy Dosing		
	N. 1015	Half 294	Full 709	High 12
**Adequate response G17 >7, n.,** **%**	354 (34.9)	85 (28.9)	261 (36.8)	8 (66.7)
**Low response G17 1-7, n.,** **%**	417 (41.1)	139 (47.3)	277 (39.1)	1 (8.3)
**No response G17 < 1, n.,** **%**	207 (20.4)	65 (22.1)	141 (19.9)	1 (8.3)
**CAG, n.,** **%**	37 (3.6)	5 (1.7)	30 (4.2)	2 (16.7)

CAG: chronic atrophic gastritis; G17: gastrin 17; PPI: proton pump inhibitors.

**Table 3:** Gastric functional status (with determination of PGI, G17, PGII, and PGI/PGII ratio) according to response rate to PPI therapy, classified according to elevation of G17 levels.

	Gastric Functional Status			
	**PG-I (μg/L)** **Mean ± DS**	**G-17 (pmol/L)** **Mean ± DS**	**PG-II (pmol/L)** **Mean ± DS**	**PGI/PGII Ratio**
**Total**	139.1 ± 97.3	**11.7 ± 21.1**	**12.8 ± 10.7**	**12.4 ± 5.1**
**Adequate response G-17 > 7**	193.9 ± 120.8	**21.8 ± 17.5**	**18.2 ± 13.6**	**12.6 ± 5.5**
**Low response G-17 1-7**	127.1 ± 68.5	**3.1 ± 1.73**	**11.1 ± 8.5**	**12.9 ± 4.6**
**No response G-17 < 1**	91.2 ± 40.9	**0.38 ± 0.29**	**7.52 ± 3.8**	**12.9 ± 3.9**
**CAG**	17.0 ± 16.7	**73.7 ± 54.2**	**8.6 ± 4.4**	**1.95 ± 1.8**

CAG: chronic atrophic gastritis; G17: gastrin 17; PG-I: pepsinogen I; PG-II: pepsinogen II.

**Table 4:** Logistic regression analysis for individual factors influencing the therapeutic response to PPI.

	Therapeutic Response to PPIs		
	OR	(95% CI)	*p*
Older Age (>60 years)	1.419	(1.070–1.882)	0.002
Gender (F)	1.015	(1.006–1.025)	0.015
Previous HP eradication	0.896	(0.628–1.276)	0.541
Active HP infection	1.578	(1.135–2.194)	0.007
Branded PPIs	1.407	(1.067–1.855)	0.0015
Full Dosage PPIs	1.712	(1.276–2.298)	0.0001

HP: *Helicobacter pylori*; PPIs: proton pump inhibitors.

**Table 5:** Employed dosing of proton pump inhibitors (PPIs) [24,25].

	Dose		
**Molecule**	**Half**	**Full**	**High**
**Omeprazole**	10 mg/day	20 mg/day	40 mg/day
**Lansoprazole**	15 mg/day	30 mg/day	60 mg/day
**Pantoprazole**	20 mg/day	40 mg/day	80 mg/day
**Rabeprazole**	10 mg/day	20 mg/day	40 mg/day
**Esomeprazole**	20 mg/day	40 mg/day	80 mg/day

## Data Availability

Not applicable.

## Supplementary Materials

The following supporting information can be downloaded at: https://www.mdpi.com/article/10.3390/ijms24032378/s1.

## Abbreviations

CAG	chronic atrophic gastritis
PG-I	Pepsinogen I
PG-II	Pepsinogen II
G17	gastrin 17
HP	Helicobacter pylori
NSAIDs	Non-Steroidal Anti-inflammatory Drugs
PPIs	proton pump inhibitors
µg	micrograms
pmol	picomoles
U/L	Unit/liter
