# Supplementary material for: Appropriateness of Proton Pump Inhibitor Prescription Evaluated by Using Serological Markers
Source: Int J Mol Sci. 2023 Jan 25;24(3):2378. doi: 10.3390/ijms24032378 (PMC9917011; doi:10.3390/ijms24032378)
Supplement: Supplementary file 1 [file ijms-24-02378-s001.zip › ijms-2095222-supplementary.pdf]

Supplementary Materials

Serological evaluation of gastric function

In order to establish the concentration of prostaglandins (PGs) and gastrin 17 (G-17) a series of processes were performed, according to the Manufacturer's instructions (Biohit (GastroPanel®, Biohit Oyj, Helsinki, Finland):

- a PGI, PGII or G-17 specific monoclonal autoantibody was fixed on the microplate;
- the serum obtained from the blood sample was deposited in the well;
- the well underwent washing in order to remove the residuals of samples or other protein fraction nonspecific links;
- a PGs antibody peroxidase-conjugate was added to the well; a chromatic effect was produced;
- a stop solution was added to stop the enzymatic reaction.
- PG and G 17 concentrations were determined using a spectrophotometer and expressed in µg/L.

For the diagnosis of *Helicobacter pylori* infection, serum was added to the well containing the specific antigen for the antibody and after washing, the marked antibody was added to bind the antigen-antibody complex. The use of the substrate for the enzyme permitted to define the absorbance.

Endoscopic and histological diagnosis

According to the protocol, gastric biopsies included: 2 samples from the antrum, 1 from the incisura angularis, and 2 from the corpus.

Histological analysis, performed according to the Operative Link on Gastritis Assessment (OLGA staging system) considered the following parameters reduction of the number of oxyntic glands or the metaplastic transformation of the cells, both leading to loss of appropriate glands and loss of hydrochloric acid output.

The atrophy was classified as mild when the rate fibrosis/metaplasia was between 0 and 30%, moderate 31-60% and severe above 61%. From the evaluation of the different biopsies in the different locations it was possible to obtain a score of 4 degrees of severity, which correlates with the progressively increasing risk of developing gastric cancer.

Table S1 : Endoscopic findings (A) and histological diagnosis (B) in patients with chronic atrophic gastritis (CAG) and active Helicobacter pylori (HP) infection. Histological grading of atrophy is classified according to the Operative Link on Gastritis Assessment (OLGA staging) system.

A)

**Endoscopic finding**	**CAG**<br>**n. 38**<br>**N (%)**	**HP infection**<br>**n. 206**<br>**N (%)**
Normal	10 (26,3)	28(13,6)
Hiatal hernia	1 (2,6	8 (3,9)
Erosive esophagitis	0 (0)	16 (7,8)

Barrett's esophagus	0 (0)	3 (1,5)
Non-erosive, mild gastritis	12 (38,0)	93 (45,1)
Erosive gastritis	0 (0)	38 (18,4)
Gastric /Duodenal ulcer	0 (0)	9 (4,4)
Erosive duodenitis	0 (0)	5 (2,4)
Gastric (cystic) polyps	0 (0)	6 (2,9)
Atrophic gastritis	15 (39,5)	0 (0,0)

B)

Histological diagnosis according to the OLGA staging system		CAG<br>n. 38<br>N (%)	HP infection<br>n. 206<br>N (%)
Stage 0	A0C0	0 (0)	146 (70,9)
Stage 1	A1C0	0 (0)	50 (24,3)
	A0C1	1 (2,6)	0 (0)
Stage 2	A0C3	24 (63,2)	0 (0)
	A1C2	6 (15,8)	0 (0)
	A2C0	0 (0)	7 (3,4)
Stage 3	A3C0	0 (0)	3 (1,5)
	A1C3	6 (15,8)	0 (0)
Stage 4	A2C3	1 (2,6)	0 (0)
